# Comment on “Aggregation Interface and Rigid Spots Sustain the Stable Framework of a Thermophilic *N*-Demethylase”

**DOI:** 10.1021/acs.jafc.3c07043

**Published:** 2023-12-01

**Authors:** Daniel M. Wade, Walker S. Lewis, Jonghoon Kang

**Affiliations:** Department of Biology, Valdosta State University, Valdosta, Georgia 31698, United States

## Abstract

The thermal properties of proteins are very important in industrial, agricultural, and food chemistry. A recent article (LiB., et al. J. Agric. Food Chem.2023, 71, 5614−562937000489 10.1021/acs.jafc.3c00877) examines the thermal denaturation of enzymes TrSOX and BSOX by measuring the enthalpy change and melting temperature in the denaturation. In this work, we report the numerical values of entropy in the denaturation of proteins and show that both proteins TrSOX and BSOX exhibit enthalpy–entropy compensation in thermal denaturation, which results in a limited variation of melting temperature in both proteins. Our analysis may serve to improve our understanding of thermal properties in proteins in food chemistry.

In a recent publication in the *Journal of Agricultural and Food Chemistry*, Li et al.^1^ used diverse biochemical and biophysical methods to examine the nature of the high thermostability of a *N*-demethylase from thermophilic *Thermomicrobium roseum*, compared with that from mesophilic *Bacillus subtilis*. The *N*-demethylase they examined in the paper was sarcosine oxidase; the one from *T. roseum* was denoted TrSOX, while that from *B. subtilis* was denoted BSOX.^1^ The authors examined the molecular basis of the high thermostability of TrSOX using several techniques, including thermodynamic analysis of the thermal denaturation of proteins. This is notably motivating, as evidenced by the increasing prominence of thermodynamics in the field of food science, as reflected in recent scholarly publications.^2−4^ One of the main results of the research is that the melting temperature (*T*_m_) for all variants (wild type and mutants) of TrSOX was significantly higher than those of BSOX. They then discussed this phenomenon in terms of denaturation enthalpy (Δ*H*). Although both Δ*H* and *T*_m_ offer valuable insights into the thermodynamic underpinnings of the thermal stability of proteins, the original paper does not address another essential thermodynamic parameter, the entropy of denaturation (Δ*S*). Δ*S* is regarded as being crucial for comprehending the forces involved in protein denaturation.^5−10^ In this Correspondence, we present our examination of their findings, aiming to elucidate the Δ*S* values in the thermal denaturation of the proteins, their correlation with Δ*H*, and the potential implications of the relationship between Δ*H* and Δ*S* within the context of the thermal stability of proteins.

Given that protein denaturation represents a phase transition from the native state to the denatured state, Δ*S* can be computed using the following equation:
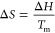
1where *T*_m_ is the melting temperature in kelvin.^11^ Numerical values of both Δ*H* and *T*_m_ for the 55 denaturation reactions obtained from the original paper,^1^ 34 for TrSOX and 21 for BSOX, were used for the calculation of Δ*S* using eq 1. Figure 1A displays the resulting values of Δ*S* and the corresponding Δ*H* values. The plot clearly shows three thermodynamic characteristics in the denaturation of the proteins. First, the denaturation of both TrSOX and BSOX at the melting temperature is an entropy-driven process; in other words, Δ*S* > 0. Second, site-directed mutagenesis decreases both Δ*H* and Δ*S* in the case of TrSOX but increases them in the case of BSOX, in most cases. Third, linear regression using eq 2 indicates a highly significant correlation between Δ*H* and Δ*S* in both proteins as the coefficients of determination (*R*^2^) are 0.9966 and 0.9992 for TrSOX and BSOX, respectively:
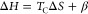
2where *T*_C_, the compensation temperature, is the slope of the fitting line^12^ and β is the *y*-intercept (Figure 1A). The strong correlation between Δ*H* and Δ*S* is known as enthalpy–entropy compensation, often observed in a weakly coupled system.^13−16^Figure 1A clearly indicates that the denaturation of TrSOX and BSOX exhibits compensatory behavior, suggesting that the molecular components responsible for the denaturation of those proteins exhibit the property of a weakly coupled system. The compensation means that as Δ*H* increases the corresponding Δ*S* also increases so that the resulting differences in *T*_m_ are minimized.^10^

**Figure 1:**
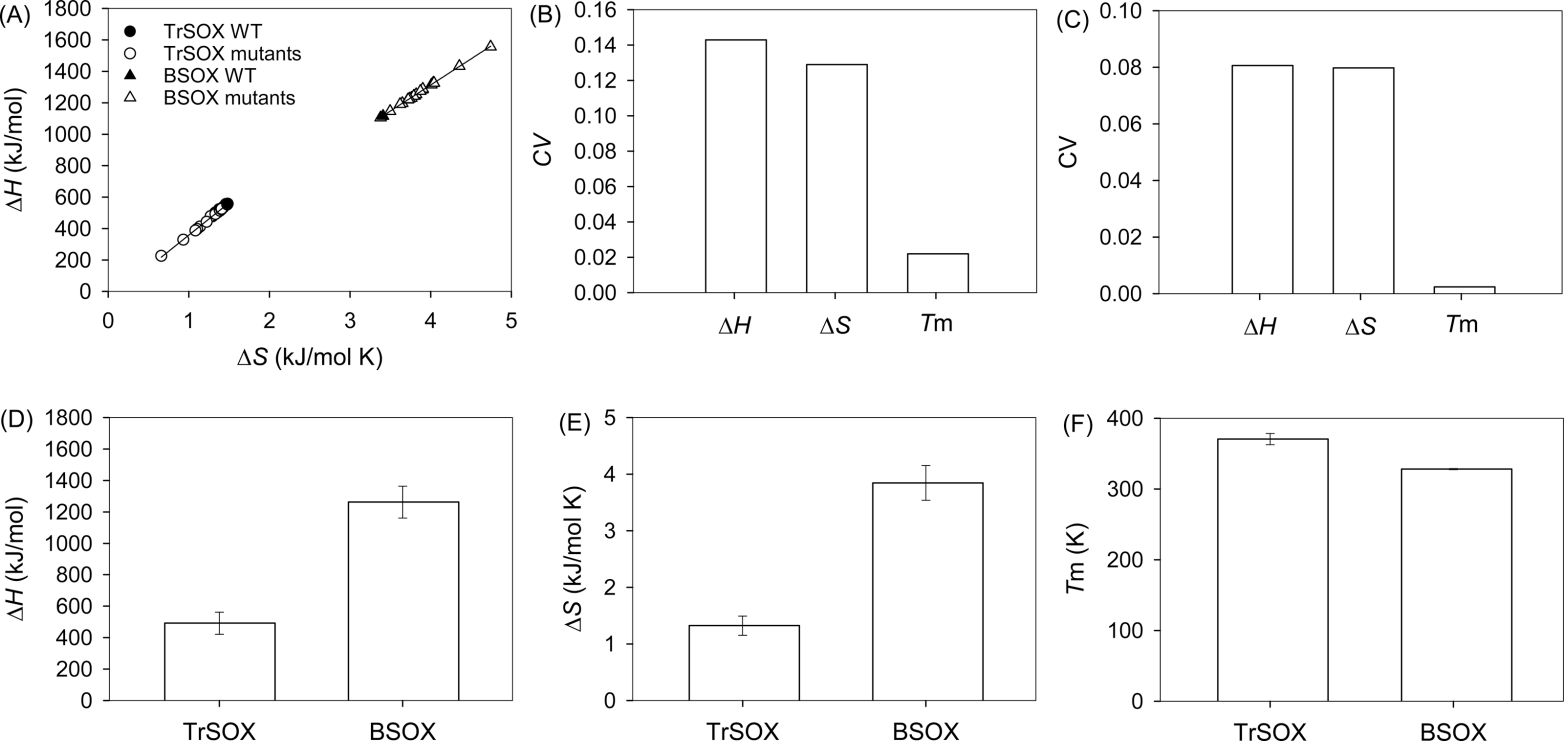
Statistical analysis of the thermodynamic parameters of TrSOX and BSOX. (A) Enthalpy–entropy compensation for both TrSOX and BSOX with wild types marked separately. Coefficients of variation of Δ*H*, Δ*S*, and *T*_m_ for (B) TrSOX and (C) BSOX. Average values and standard deviations of (D) Δ*H*, (E) Δ*S*, and (F) *T*_m_ for TrSOX and BSOX. SigmaPlot (version 15, Systat Software Inc., San Jose, CA) was used for graph preparation and statistical analysis.

The values of *T*_C_ and its standard errors for TrSOX and BSOX are 411.6 ± 4.2 and 331.6 ± 2.1 K, respectively. A Student’s *t* test indicates that the difference in *T*_C_ between TrSOX and BSOX is statistically significant (Table 1). In the *t* test, the degree of freedom (df)^17^ was calculated as df = (*n*_1_ – 2) + (*n*_2_ – 2), where *n*_1_ and *n*_2_ are the number of data points of TrSOX and BSOX, respectively: *n*_1_ = 34, and *n*_2_ = 21 (Figure 1A). *T*_C_ can quantitatively measure the degree of compensation between Δ*H* and Δ*S*.^12^ The statistical difference in *T*_C_ between TrSOX and BSOX (Table 1) strongly suggests that denaturation of each protein follows a distinct mechanism.

**Table 1:** Statistical Comparison of the Thermodynamic Parameters between TrSOX and BSOX

	*T*_C_	Δ*H*	Δ*S*	*T*_m_
df	51	53	53	53
*t*	14.1	–33.3	–39.3	24.2
*p*	3.4 × 10^–19^	3.4 × 10^–37^	7.3 × 10^–41^	2.5 × 10^–30^

The compensatory tendencies of Δ*H* and Δ*S* can be quantitatively described by comparing the coefficient of variation (CV) for each thermodynamic parameter, as determined by eq 3:
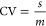
3where *s* and *m* are the standard deviation and the mean of the samples, respectively.^17^ The CVs of Δ*H* and Δ*S* are more than 5 or 33 times larger than that of *T*_m_ for TrSOX (Figure 1B) or BSOX (Figure 1C), respectively. On the basis of this result, we suggest that variations in Δ*H* and Δ*S* are local characteristics for the structural thermodynamics of proteins, while variation in *T*_m_ is a global characteristic that stays relatively constant in a weakly coupled system. This thermodynamic explanation is in line with the observations in the original paper^1^ that all 20 mutants of BSOX generated with an intention to improve its thermostability showed a large variation in both Δ*H* and Δ*S* but a highly limited variation in *T*_m_.

We also compare the thermodynamic parameters of TrSOX and BSOX to elucidate thermodynamic reasons for the high thermal stability of TrSOX. The differences in Δ*H* (Figure 1D), Δ*S* (Figure 1E), and *T*_m_ (Figure 1F) are shown to be statistically significant on the basis of the *p* values (Table 1). While Δ*H* is much smaller in TrSOX suggesting TrSOX must have a smaller value of *T*_m_ according to eq 1, Δ*S* is also much smaller in TrSOX, making it more stable. The much smaller value of Δ*S* stabilizes TrSOX compared to BSOX. In other words, the high thermal stability of TrSOX can be explained by the small value of Δ*S*. This is why Δ*H* is not sufficient in the explanation of the variation of *T*_m_ and Δ*S* should be included in the interpretation. The analysis introduced in this paper can be applied to other thermostable proteins such as TrLipB^18,19^ to assess the contribution of small values of denaturation entropy to the thermal stability of proteins. We can conclude that the thermal denaturation of both TrSOX and BSOX exhibits enthalpy–entropy compensation. Statistical analysis suggests that Δ*S* is responsible for the high thermal stability of TrSOX. It will be interesting to examine whether other thermostable proteins exhibit these phenomena.
